# The multifaceted roles of apolipoprotein E4 in Alzheimer’s disease pathology and potential therapeutic strategies

**DOI:** 10.1038/s41420-025-02600-y

**Published:** 2025-07-08

**Authors:** Yongfeng Chen, Haiping Jin, Jia Chen, Jing Li, Mihnea-Alexandru Găman, Zhenyou Zou

**Affiliations:** 1 School of Medicine, Taizhou University, Taizhou 318000, Zhejiang China; 2 Taizhou Hospital, Taizhou 317000, Zhejiang China; 3 School of Medicine, Zhejiang University, Hangzhou 310000, Zhejiang China; 4 Institute of Basic Medicine, North Sichuan Medical College, Nanchong 637000, Sichuan China; 5 Faculty of Medicine, “Carol Davila” University of Medicine and Pharmacy, Bucharest, 050474 Romania; 6 Department of Cellular and Molecular Pathology, Stefan S. Nicolau Institute of Virology, Romanian Academy, Bucharest, 030304 Romania; 7 Department of Hematology, Center of Hematology and Bone Marrow Transplantation, Fundeni Clinical Institute, Bucharest, 022328 Romania; 8 Liuzhou Key Lab of Psychosis Treatment, Brain Hospital of Guangxi Zhuang Autonomous Region, Liuzhou 545005, Guangxi China; 9 Osaka Metropolitan University Graduate School of Medicine, Osaka, 545-8585 Japan

**Keywords:** Cell signalling, Cognitive ageing

## Abstract

Alzheimer’s disease (AD), the most common dementia in the elderly, is marked by progressive cognitive decline and neurodegeneration. Core pathological hallmarks include amyloid-beta (Aβ) plaques, hyperphosphorylated tau aggregates, neuroinflammation, and metabolic dysfunction (e.g., impaired glucose utilization, mitochondrial deficits). Apolipoprotein E4 (ApoE4), the strongest genetic risk factor for AD, interacts with these processes, yet its precise pathogenic mechanisms remain unclear. This review examines ApoE4’s multifaceted contributions to AD pathogenesis, focusing on its roles in Aβ accumulation, tau hyperphosphorylation, neuroinflammatory activation, and metabolic dysregulation. We further evaluate emerging therapeutic strategies targeting these pathways, including ApoE4 modulation, anti-amyloid/tau interventions, and metabolic rescue approaches. Elucidating the molecular interplay between ApoE4 and AD pathology is critical for developing targeted therapies to modify disease progression and mitigate cognitive decline in patients.

## Facts

- ApoE4 exacerbates Aβ pathology by enhancing Aβ production and impairing Aβ clearance mechanisms. ApoE4-induced metabolic dysregulation, oxidative stress, and inflammatory responses further amplify these effects.
- ApoE4 enhances tau pathology by activating oxidative stress, disrupting lipid metabolism, and triggering inflammatory signaling.
- ApoE4 increases ROS production, activates the NLRP3 inflammasome, and modulates the TREM2 signaling pathway to amplify microglial inflammation.
- ApoE4 disrupts neuronal energy metabolism and mitochondrial function by competing for insulin receptors, inhibiting key metabolic enzymes, and promoting lipid accumulation.

## Open questions

- What are the precise molecular mechanisms by which ApoE4 disrupts Aβ clearance and accelerates its production?
- How does ApoE4 specifically alter tau pathology through oxidative stress and lipid metabolism in neurons and glial cells?
- What therapeutic strategies can effectively target ApoE4-induced microglial inflammation and improve neuroinflammation in Alzheimer’s disease?

## Introduction

Alzheimer’s disease (AD) is a neurodegenerative disorder marked by progressive cognitive decline and memory loss, representing the most common form of dementia in the elderly. While the precise pathogenesis of AD remains elusive, research indicates that key pathological features include the accumulation of extracellular amyloid-beta (Aβ) plaques, abnormal tau phosphorylation and aggregation, neuroinflammation, and various metabolic disruptions, such as impaired glucose metabolism and mitochondrial dysfunction. These factors are interrelated, contributing to the progression of neurodegeneration [1].

Apolipoprotein E (ApoE), an important lipoprotein in the central nervous system, is crucial for lipid transport and cholesterol homeostasis in the brain [1]. It is mainly produced by astrocytes and activated microglia in the brain, although recent studies have also demonstrated ApoE production by neurons [1–3]. In peripheral tissues, it is synthesized by the liver and macrophages [3]. The human ApoE gene has three isoforms—ApoE2, ApoE3, and ApoE4—of which ApoE4 is the major genetic risk factor for AD. Despite minor structural differences between ApoE4 and the other isoforms, ApoE4 is more compact and unstable, which affects its lipid-binding ability and impairs the clearance of amyloid-beta degradation products [4]. Furthermore, ApoE4 is implicated in hyperphosphorylation of tau, enhanced neuroinflammation, and increased oxidative stress [5].

The pathological effects of ApoE4 also extend to disruptions in brain energy metabolism. ApoE4 carriers exhibit impaired mitochondrial function, increased lipid oxidation, and interference with insulin signaling, leading to early-stage brain glucose metabolism deficits. These metabolic abnormalities significantly increase the risk of cognitive decline in ApoE4 carriers [6].

This review explores the mechanisms by which ApoE4 influences Aβ accumulation, tau phosphorylation, neuroinflammation, and metabolic dysregulation, and evaluates current therapeutic strategies targeting these processes. By understanding the multifaceted roles of ApoE4, we aim to provide new insights for AD research and support future clinical treatments and personalized medicine.

## Mechanisms underlying ApoE4 regulation of Aβ pathology

One of the key pathological features of Alzheimer’s disease (AD) is the extracellular deposition of Aβ in the brain in the form of amyloid fibrils. Aβ is a 38- to 43-residue peptide fragment derived from the membrane-bound β-amyloid precursor protein (APP) *via* sequential proteolytic cleavages by β- and γ-secretase enzyme activities [4]. The Aβ protein includes two major forms, Aβ42 and Aβ40, with Aβ42 being more toxic and more prone to aggregation [7]. Aβ aggregates, including oligomeric aggregates (oAβ) and amyloid plaques, can interfere with normal neuronal function in various ways, including damaging cell membranes, disrupting intracellular signaling, inducing oxidative stress, and promoting inflammation [8].

Although ApoE’s critical role in AD pathology is well established, the exact mechanisms by which it influences disease progression remain unclear. ApoE may act both as an AD inhibitor and promoter [4, 9], profoundly affecting Aβ metabolism through several pathways: (1) Promoting APP Expression: ApoE4 enhances APP transcription directly or via signaling pathways like extracellular signal-regulated kinase (ERK), nuclear factor-κB (NF-κB), and C/EBPβ [10, 11]. ApoE4 could also boost APP transcription by interacting with transcription factors like Sp1 and through epigenetic modifications that alter the chromatin structure of the APP gene [12, 13]. (2) Enhancing APP Processing Enzyme Activity: ApoE4 increases BACE1 transcription through interactions with transcription factors such as NF-κB, C/EBPβ, and Sp1 [11, 12], and promotes BACE1 activity by inhibiting insulin signaling and activating GSK3β [14]. ApoE’s C-terminal region can inhibit γ-secretase in neurons, providing a protective effect against Aβ generation, though this inhibition is weaker in ApoE4 [15]. (3) Cholesterol Metabolism Disruption: ApoE4 increases cholesterol secretion from astrocytes, which enhances lipid raft formation in neurons, aiding APP localization and processing [16]. High cholesterol levels promote APP transport to lipid rafts, where it interacts with β- and γ-secretase, driving Aβ production [17]. (4) Endosomal Trafficking Abnormalities: ApoE4 promotes APP internalization and colocalizes APP and BACE1 in early endosomes, delaying endosomal-lysosomal trafficking and APP processing, thus promoting Aβ generation [18–20]. In addition to these pathways, ApoE4 mediates inflammatory responses, metal ion dysregulation, oxidative stress, and energy metabolism defects, all of which may contribute to Aβ production and aggregation [21–24].

Aβ is primarily produced in neurons and secreted into the interstitial fluid (ISF). Proteolytic degradation by endopeptidases (e.g., insulin-degrading enzyme (IDE), neprilysin (NEP)) constitutes a major Aβ clearance pathway [25]. Studies have shown that NEP and IDE are expressed in both neurons and glial cells, while ApoE4 can inhibit the expression and activity of IDE and NEP, thereby preventing Aβ clearance [26, 27]. It has been reported that the expression of IDE and NEP in the brains of ApoEε4 carriers is significantly lower compared to non-carriers [26, 28, 29]. In neurons, ApoE4 can downregulate IDE expression by activating the N-methyl-D-aspartate (NMDA)/cAMP-dependent protein kinase (PKA) signaling pathway [30]. Studies on astrocytes have shown that under LPS signaling stimulation, ApoE4 can downregulate NEP expression via TLR4-dependent pathways, and the use of LXR agonists can promote NEP upregulation. This suggests that the impact of ApoE4 on NEP expression may be related to inflammatory responses and cholesterol metabolism dysregulation [31].

**Fig. 1:**
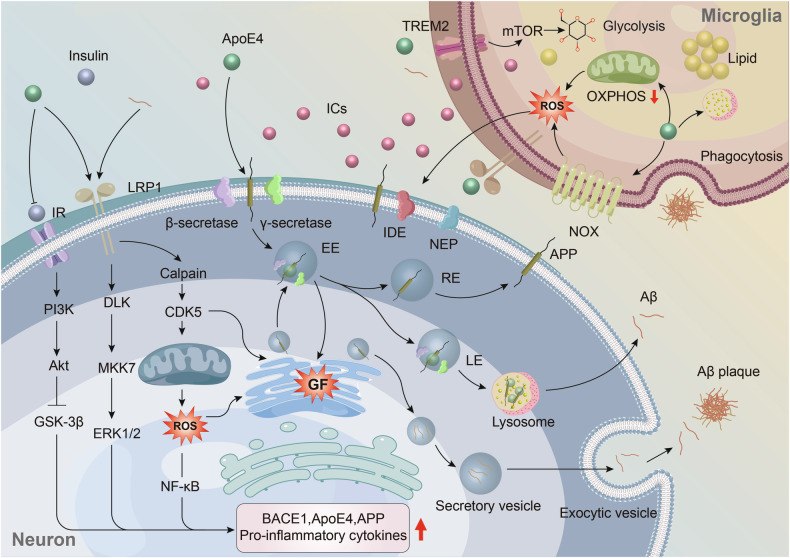
Possible mechanisms by which ApoE4 affects Aβ production and clearance. 1.In the context of ApoE4, microglia exhibit pathological features such as lipid accumulation, increased ROS production, elevated ICs secretion, and lysosomal dysfunction, which impair their phagocytic function and effective clearance of Aβ [36, 94, 129]. ROS reduce the activity of Aβ-degrading enzymes, such as IDE and NEP [130, 131], while inflammatory cytokines promote the expression of APP as well as β- and γ-secretases, thereby enhancing APP processing [132, 133]. 2.ApoE4 may drive microglia to shift from OXPHOS to aerobic glycolysis to meet the high energy demands of the inflammatory respons. In this process, the TREM2/mTOR pathway plays a crucial role [39]. However, ApoE4 may negatively regulate TREM2 signaling [95, 97]. Furthermore, ApoE4 could interfere with Aβ uptake and internalization by competing with Aβ for receptors like LRP1 and LDLR [32, 33]. 3.ApoE4 can activate ERK and NF-κB signaling and inhibit insulin signaling, promoting the transcription of APP and BACE1 [10, 28, 57, 134]. 4.ApoE4 may act as a molecular chaperone to facilitate the internalization of APP and promote the co-localization of APP and BACE1 in EE, while delaying endosome-lysosome recycling [18–20]. 5.Both ApoE4 and Aβ can activate CDK5 signaling or increase ROS production, leading to GF, which increases the co-localization of APP and BACE1 and promotes Aβ generation [53, 135]. AKT, protein Kinase B; APP, β-amyloid precursor protein; BACE1, Beta-site amyloid precursor protein cleaving enzyme 1; CDK5, cycline dependent kinase-5; DLK, dual leucine-zipper kinase; EE, early endosome; ERK1/2, extracellular signal-regulated kinase 1/2; GF, golgi fragmentation; GSK-3β, glycogen synthase kinase-3β; ICs, inflammatory cytokines; IDE, insulin-degrading enzyme; IR, insulin receptor; LDLR, low-density lipoprotein receptor; LE, late endosome; MKK7, mitogen-activated protein kinase kinase 7; LRP1, low-density lipoprotein receptor-related protein 1; mTOR, mechanistic target of rapamycin; NEP, neprilysin; NOX, NADPH oxidase; OXPHOS, oxidative phosphorylation; PI3K, phosphoinositide 3-kinase; RE, recycle endosome; ROS, reactive oxygen species; Trem2, Triggering Receptor Expressed on Myeloid Cells 2.

Cellular clearance of Aβ, mediated by neurons, microglia, and astrocytes through the lysosomal system, plays a key role in Aβ degradation [25]. Both ApoE and Aβ bind to receptors such as LRP1, LDLR, and HSPGs on neurons and glial cells, meaning ApoE can compete with Aβ for receptor binding, potentially interfering with Aβ uptake and clearance [32, 33]. ApoE4 exacerbates this by impairing the autophagy-lysosome pathway, downregulating autophagy-related genes, inducing lysosomal alkalization, and disrupting lysosomal membrane integrity, all of which hinder Aβ degradation [20, 34, 35]. Furthermore, ApoE4 reduces cholesterol efflux, leading to cholesterol accumulation inside cells, which decreases Rab7 recycling. This slows Aβ transport to the lysosome [36]. Cholesterol buildup in the late endosome-lysosome compartment also raises their pH, impairing function and further obstructing Aβ clearance [37]. On the other hand, ApoE4-induced mitochondrial dysfunction and insulin resistance negatively affect energy metabolism, promoting BACE1 expression and compromising Aβ clearance [24, 38]. Studies show that in response to Aβ, microglia shift from oxidative phosphorylation (OXPHOS) to glycolysis to meet energy demands for proliferation, migration, cytokine secretion, and phagocytosis. However, widespread metabolic defects in both glycolysis and OXPHOS push microglia into a chronic tolerance state, reducing immune responses, including diminished cytokine secretion and phagocytosis [39] (Fig. 1).

Currently FDA-approved Aβ-targeting drugs include Aducanumab and Lecanemab. While Aducanumab slows AD progression by reducing Aβ plaques, its cognitive benefits remain controversial, and some patients may experience side effects such as brain edema [40]. Lecanemab, in clinical trials, has demonstrated efficacy in slowing cognitive decline in patients with mild AD and significantly reducing Aβ deposition, though challenges remain in fully addressing both clinical efficacy and side effects [41]. Besides, several anti-amyloid antibodies currently under investigation, such as Crenezumab and Gantenerumab, have also shown great potential in clearing Aβ. However, these antibodies generally carry a significant risk of amyloid-related imaging abnormalities (ARIA), particularly in ApoE4 carriers, which limits their use [42]. At present, new immunotherapies and vaccines are also under development, aiming to eliminate Aβ deposition through more precise immune responses (Table 1).

**Table 1:** Overview of Aβ-targeted drugs.

Drug name	Drug type	Clinical effects	Clinical stage	Ref.
Aducanumab	Monoclonal Antibody	Reduces Aβ plaques in early AD, but clinical effects on cognition are controversial	Approved (FDA)	[142, 143]
Lecanemab	Monoclonal Antibody	Moderately reduces cognitive decline and amyloid burden in early AD	Approved (FDA)	[144]
Solanezumab	Monoclonal Antibody	No significant effect on cognitive decline in preclinical AD	Phase III Clinical Trials	[145]
Crenezumab	Monoclonal Antibody	Well tolerated but did not reduce clinical decline in participants with early AD	Phase III Clinical Trials	[146]
Gantenerumab	Monoclonal Antibody	Limited effect on cognition improvement, long-term effects remain uncertain	Phase III Clinical Trials	[147]
ACC-001	Vaccine	Shows immune response but no significant cognitive improvement	Phase II Clinical Trials	[148, 149]

It is noteworthy that both AD and cerebral amyloid angiopathy (CAA) are characterized by the accumulation of Aβ in the brain. However, while Aβ primarily deposits in the brain parenchyma in AD, in CAA, it mainly deposits in the walls of cerebral blood vessels [43]. CAA is commonly observed in patients with AD. Genetically, AD and CAA share the ApoEε4 gene as one of the strongest genetic risk factors for both diseases. This shared genetic risk may contribute to vascular damage in CAA during anti-Aβ antibody treatments, which manifests as amyloid-related imaging abnormalities (ARIA) [43, 44]. Current epidemiological studies suggest that the ApoEε2 allele may promote specific ‘CAA-related vascular changes’ and potentially act as a risk factor for amyloid-related vascular hemorrhage. In contrast, ApoEε4 primarily enhances Aβ deposition in the walls of cerebral blood vessels, with oxidative stress potentially playing a key role in this process [45, 46]. In the 5XFAD mouse model, which expresses human ApoE4+/+ (5XE4) and exhibits prominent CAA and parenchymal plaque pathology, treatment with anti-Aβ antibodies exacerbated the severity of microhemorrhages. However, treatment with the anti-human ApoE antibody HAE-4 effectively reduced Aβ deposition [44]. These findings suggest that the risks associated with CAA and the complex role of ApoE in anti-Aβ treatment for AD should be considered, highlighting the importance of personalized treatment strategies.

## Mechanisms underlying ApoE4 regulation of tau pathology

Phosphorylated tau (p-tau) is another hallmark of Alzheimer’s disease, alongside Aβ plaques. Tau, a protein mainly found in neuronal axons, stabilizes microtubules essential for maintaining neuronal structure and function. Under normal conditions, moderately phosphorylated tau binds to microtubules and lipid membranes, supporting neuronal integrity and signaling. However, excessive phosphorylation impairs tau’s binding to microtubules, destabilizing the cytoskeleton, leading to neurofibrillary tangles (NFTs) and disrupting normal neuronal function [47]. For a long time, the prevailing view has been that the abnormal accumulation of Aβ and p-tau are interrelated in the pathology of AD [48]. However, several studies have pointed out that these two pathologies may be independent of each other [49, 50]. Specifically, research by Wang et al. showed that in human neurons derived from induced pluripotent stem cells (hiPSC) expressing ApoE4, p-tau levels were abnormally elevated even when Aβ generation was blocked, specifically leading to degeneration and loss of GABAergic neurons. This finding suggests that ApoE4 may independently promote tau pathology through mechanisms unrelated to Aβ pathology [49]. In P301S Tau transgenic mice carrying different ApoE isoforms, tau protein-mediated neurodegeneration exhibited an ApoE isoform-dependent pattern (ApoE4 > ApoE3 > ApoE2), and the deletion of ApoE4 significantly reduced tau pathology, implying that ApoE4 may participate in the progression of tau pathology through a toxic gain-of-function mechanism [50]. It has been reported that knocking down ApoE4 in astrocytes can rescue tau pathology and microglial phagocytosis of synaptic material [51]. However, other studies have shown that human astrocyte-derived ApoE4 does not confer detrimental effects in human neurons (at least with regard to tau phosphorylation, GABAergic neuron degeneration, and Aβ production) [49]. In tau mouse models, selective knockout of ApoEε4 in neurons led to significant improvements in tau pathology, glial proliferation, neurodegeneration, neuronal hyperexcitability, and myelin loss [52]. These findings suggest that the toxic effects of ApoE4 may be neuron-specific. The specific toxic role of ApoE4 from different cell sources in tau pathology requires further investigation to provide a comprehensive understanding.

The exact mechanisms by which ApoE4 regulates tau pathology are not yet fully understood, but several possible pathways have been proposed based on current research: (1) Imbalance in Tau Kinases/Phosphatases System: ApoE4 can promote tau phosphorylation by activating several kinases, such as glycogen synthase kinase-3β (GSK-3β), cyclin-dependent kinase-5 (CDK5), and mitogen-activated protein kinase (MAPK) [53–55]. On the other hand, ApoE4 can influence tau dephosphorylation by inhibiting the activity of the major tau phosphatase protein phosphatase 2A (PP2A) [56]. (2) Neuroinflammation: ApoE4 plays a critical role in neuroinflammation. ApoE4 can activate inflammatory signaling pathways through multiple routes, promoting the release of pro-inflammatory cytokines, which in turn activate kinases such as GSK-3β, inducing tau hyperphosphorylation and the formation of NFTs [57–59]. (3) Dysregulation of Lipid Metabolism: The inefficiency of ApoE4 in lipid transport leads to inadequate cholesterol turnover in neurons, an imbalance in specific phospholipid ratios, lipid accumulation, and increased lipid peroxidation. These changes disrupt the structure and function of cell membranes, affect tau phosphorylation-related signaling, and ultimately exacerbate tau pathology [60–62]. (4) Iron Overload: ApoE4 increases the expression of ferritin, the iron storage protein, leading to excessive intracellular iron deposition. This excess iron activates kinases associated with tau phosphorylation by promoting oxidative stress (OS) and other signaling pathways. Severe OS can even trigger ferroptosis [63, 64]. (5) Oxidative Stress: ApoE4 can impair mitochondrial function through various mechanisms, leading to increased ROS generation and triggering OS [65, 66]. OS not only activates kinases such as GSK-3β and CDK5 but also promotes tau phosphorylation through mechanisms such as inducing inflammation, inhibiting insulin secretion, and altering tau protein conformation [65–68].

**Fig. 2:**
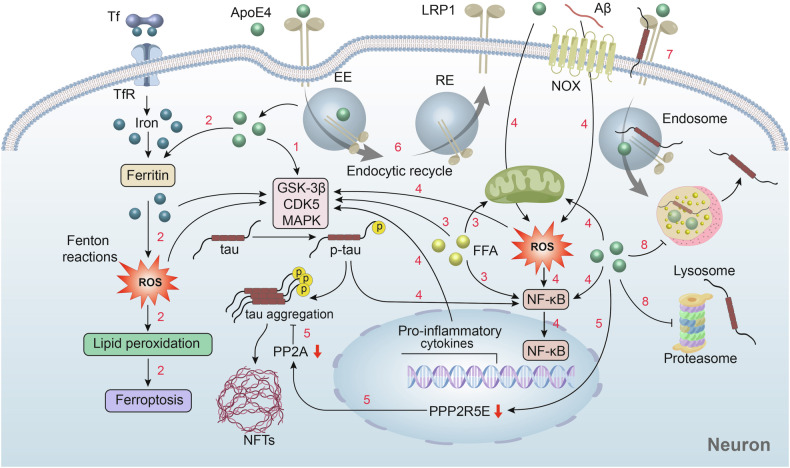
Possible mechanisms by which ApoE4 affects tau phosphorylation and clearance. 1.ApoE4 may promote tau phosphorylation by activating relevant kinases [53–55]. 2.ApoE4 can enhance the expression of ferritin, induce iron overload, trigger oxidative stress and lipid peroxidation, and even lead to ferroptosis [22, 63, 64]. 3.ApoE4-mediated lipid metabolism defects result in elevated fatty acid levels, which activate tau phosphorylation-related kinases, impair mitochondrial function, and activate the NF-κB pathway [60–62]. 4.ApoE4 can promote the generation of ROS and activate the NF-κB signaling pathway, which in turn activates kinases such as GSK-3β, leading to tau phosphorylation [65–68, 88]. Phosphorylated tau, in turn, can further activate the NF-κB pathway, exacerbating the inflammatory response [136]. 5.ApoE4 significantly downregulates the expression of the PPP2R5E gene, leading to decreased activity of the major tau phosphatase PP2A [56]. 6.ApoE4 can promote the endocytic recycling of LRP1, providing a channel for tau diffusion [75]. 7.ApoE4 competes with tau for LRP1, restricting tau diffusion [77]. 8. ApoE4 interferes with the autophagy-lysosome pathway and the ubiquitin-proteasome system (UPS), hindering tau degradation [20, 71–73]. CDK5 cycline dependent kinase-5, GSK-3β glycogen synthase kinase-3β, MAPK mitogen- activated protein kinase, FFA free fatty acid, NFTs neurofibrillary tangles, PP2A protein phosphatase 2A, Tf transferrin, TfR transferrin receptor.

Under both physiological and pathological conditions, tau protein metabolism involves two major pathways: the autophagy-lysosome pathway and the ubiquitin-proteasome system (UPS) [69]. The presence of ApoE4 has been shown to be associated with dysfunction in the autophagy-lysosome pathway. Specifically, ApoE4-expressing cells exhibit inhibited autophagy, reduced lysosomal proteolysis, insufficient lysosomal acidification, and lysosomal rupture, leading to the premature release of undigested contents, which may affect tau clearance [20, 70, 71]. Additionally, ApoE4 has been confirmed to potentially interfere with ubiquitin signaling and protein degradation processes, thereby affecting tau metabolism and aggregation [72, 73]. Recent studies have confirmed that LRP1 plays a key role in controlling tau endocytosis, degradation, and subsequent propagation [74]. ApoE4, through its cofactor glypican-4 (GPC-4), not only strongly induces tau hyperphosphorylation but also promotes the transport of LRP1 to the cell surface, providing a crucial channel for tau protein diffusion [75]. However, other studies have found that ApoE4 can reduce the distribution of receptors such as LRP1 on the cell membrane by interfering with receptor-mediated endocytic recycling through the endosome pathway [76]. Moreover, ApoE4 can also compete with tau protein for binding to LRP1, thereby slowing down LRP1-mediated tau endocytosis and degradation [77]. These findings suggest that ApoE4 provides a crucial channel for tau protein diffusion but also somewhat limits this diffusion, reflecting the complexity of ApoE4’s role in the regulation of tau uptake, degradation, and propagation (Fig. 2).

Currently, there are relatively few targeted drugs aimed at the tau protein. LMTM is a small-molecule drug designed to inhibit tau aggregation and tangling. While it has demonstrated good safety, it failed to significantly improve cognitive function in AD patients during clinical trials [78]. Ongoing drug developments, such as BIIB092 (Tilavonemab) and TPI-287, aim to precisely target tau protein to reduce its propagation or aggregation and improve cognitive function in patients (Table 2).

**Table 2:** Overview of tau-targeted drugs.

Drug name	Drug type	Clinical effects	Clinical stage	Ref.
Methylene Blue	Small Molecule	Limited cognitive improvement in some Phase III trials, safe with minimal side effects	Approved (FDA)	[150–152]
Gantenerumab	Monoclonal Antibody	Significantly reduced amyloid burden and may affect tau, but no significant effect was observed in tau PET	Phase III Clinical Trials	[153, 154]
TPI-287	Small Molecule	Tau therapeutic effects were not demonstrated. The treatment was prone to allergic reactions with low tolerability	Phase I Clinical Trials	[155]
Tilavonemab	Monoclonal Antibody	Generally well tolerated but did not demonstrate efficacy in treating patients with early AD	Phase II Clinical Trials	[156]

## Mechanisms underlying the role of ApoE4 in neuroinflammation

Neuroinflammation is one of the key drivers of neurodegenerative changes and is considered the third most important mechanism in AD pathology, following Aβ deposition and neurofibrillary tangles [79]. Neuroinflammation is instigated by the misfiring of immune cells in the central nervous system (CNS), involving microglia and astrocytes as key cell types [80]. In the early stages of AD, the activation of microglia and astrocytes may initially serve as a defense mechanism, aimed at protecting the brain by promoting tissue repair and clearing cellular debris and Aβ aggregates. However, over time, chronic inflammation can lead to synaptic loss and neuronal death, which exacerbates the progression of AD [81].

Microglia, the primary innate immune cells in the brain, play a central role in the brain’s immune response. However, in the context of AD, excessive microglial activation is not merely an inflammatory bystander effect, but likely a critical upstream mechanism. Research has shown that the interaction between microglial activation and Aβ pathology promotes tau protein propagation, eventually leading to widespread brain damage and cognitive impairment [82]. There is substantial evidence indicating that ApoE4 plays a key role in the activation of microglia and influences the risk and progression of AD through neuroinflammation [50, 83]. In vitro experiments have confirmed that microglia expressing ApoE4 exhibit a stronger immune response upon lipopolysaccharide (LPS) stimulation. When co-cultured with neurons, the levels of TNF-α significantly increase, leading to a notable decrease in neuronal viability and an increase in cell death [50]. Similar results have been observed in vivo, where ApoE4 mice showed increased glial cell activation in response to LPS injected into the lateral ventricle compared to ApoE2 and ApoE3 mice. Additionally, these ApoE4 mice displayed higher levels of IL-1β, IL-6, TNF-α secretion, and greater synaptic protein loss [84]. ApoE not only affects innate immunity but also participates in the regulation of a wider range of adaptive immune responses [85]. In tauopathy mouse models, ApoE4 significantly increases cytotoxic T cell numbers. Mechanistically, ApoE4 may recruit T cells by modulating microglial activation. The IFN-γ secreted by these T cells worsens microglial inflammation and antigen presentation, creating a feedback loop that accelerates tau pathology and neuronal damage [86].

During the immune activation of microglia, ROS play a crucial role as pro-inflammatory molecules [87]. ApoE4 and Aβ have been shown to increase ROS production through various mechanisms, including mitochondrial damage, activation of NADPH oxidase (NOX), and induction of endoplasmic reticulum stress [65, 88–90]. ROS, in turn, activate inflammatory signaling pathways such as NF-κB and MAPK, thereby promoting the expression of pro-inflammatory factors [91]. ROS can also enhance microglial sensitivity to pathological signals, thereby exacerbating neuroinflammatory responses [92]. Choi et al. examined human AD postmortem brain samples and found that, compared to age-matched controls, microglial “pro-inflammatory” (M1) marker gene expression was increased, and this effect was associated with an increase in the NOX2 p47phox subunit [93]. In vitro and in vivo experiments have also confirmed that inhibition or knockout of NOX2 significantly reduces the ability of Aβ to induce ROS in microglia and in the brains of aged mice [94]. These findings suggest that NOX2-derived ROS may play a key role in microglial activation. In addition, ApoE4, Aβ, and damage-associated molecular patterns (DAMPs) such as cell debris, ATP, and HMGB1 released after cellular damage can bind to various receptors on the surface of microglial membranes, including TREM2, microglial toll-like receptor 4 (TLR4), and CD36, thereby activating microglia (Table 3).

**Table 3:** Key Receptors Involved in Microglial Activation.

Receptors	Receptor type	Biological function	Implications in AD	Ref.
TREM2	Member of the immunoglobulin-like receptor family	Binds to ApoE4, Aβ, and HMGB1, promoting microglial activation and immune response	TREM2 deletion reduces microglial survival, impairs phagocytosis, and inhibits Aβ clearance	[157]
CD33	Member of the SIGLEC family proteins	Binds to Aβ, suppressing microglial phagocytosis and immune responses	Overexpression of CD33 limits Aβ clearance and enhances inflammation	[158]
LRP1	Member of the LDL receptor family, a large transmembrane receptor	Binds to Aβ, lipoproteins, and other ligands, regulating microglial activation and promoting Aβ clearance	LRP1 dysfunction impairs Aβ clearance and promotes chronic inflammation	[159]
P2Y Receptors	Member of the G-protein coupled receptor family	Binds to ATP and other purines, activating microglial responses to danger signals and promoting migration	Overactivation promotes excessive microglial inflammation	[160]
TLR4	Member of the Toll-like receptor family	Binds to Aβ, PAMPs, and DAMPs, activating microglial inflammation and immune responses	TLR4 activation promotes microglial inflammation and neurodegeneration in AD	[161]
LilrB3	Member of the LILR family proteins	Binds to ApoE4, promoting microglial activation and a pro-inflammatory response	LilrB3 interacts with ApoE4, driving microglial inflammation and impairing phagocytosis	[162]
RAGE	Member of the AGE receptor family	Binds to Aβ, HMGB1, and other DAMPs, activating microglial inflammation and promoting immune responses	RAGE activation enhances microglial activation and intensifies the inflammatory response and oxidative stress in the brain	[163]
CD36	Member of the scavenger receptor family	Binds to Aβ, oxidized lipids, PAMPs, and DAMPs, enhancing microglial phagocytosis and inflammatory responses	CD36 activation can enhance Aβ clearance but also promotes neuroinflammation	[164]
SCARA-1	Member of the scavenger receptor family	Binds to Aβ and apoptotic cells, promoting microglial phagocytosis and immune responses	SCARA-1 deficiency reduces Aβ clearance	[165]
Fc Receptors	Member of the immunoglobulin receptor family	Binds to immunoglobulin complexes and Aβ, promoting microglial activation and phagocytosis	Fc receptor activation enhances Aβ clearance but can also increase inflammation	[166]
Complement Receptor	Member of the complement receptor family	Bind to complement-opsonized Aβ, enhancing microglial phagocytosis and immune activation	Complement receptor activation may aid Aβ clearance but also promote neuroinflammation	[167]

*CR* Complement Receptor; *DAMPs* Damage-associated molecular patterns; *FPR* Formyl Peptide Receptor; *HMGB1* High Mobility Group Box 1; *LDL* Low-density lipoprotein; *LILR* Leukocyte immunoglobulin-like receptor; *LPS* Lipopolysaccharides; *MAC1* Macrophage-1 Antigen; *PAMPs* Pathogen-associated molecular patterns; *SIGLEC* Sialic acid-binding immunoglobulin-like lectin; *SR* Scavenger Receptor; *TLR* Toll-like Receptor; *TREM-2* Triggering Receptor Expressed on Myeloid Cells-2.

**Fig. 3:**
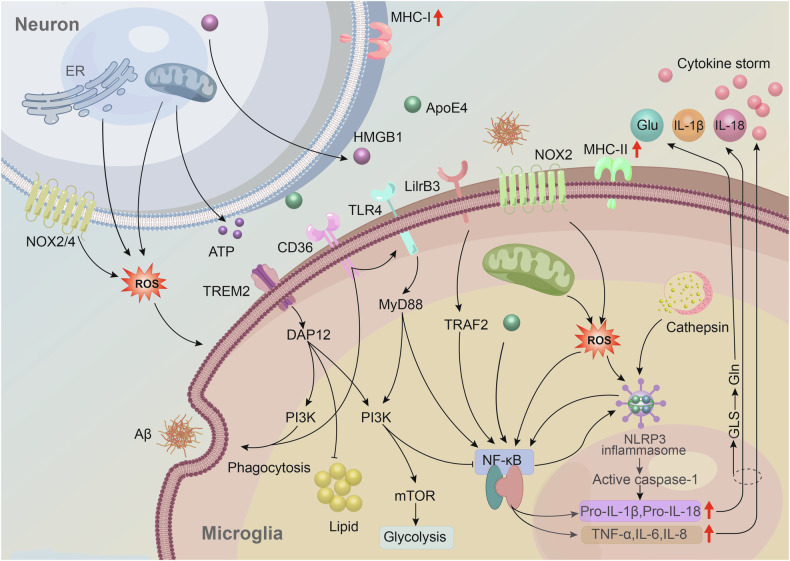
Possible mechanisms of ApoE4 in microglial immune activation. 1.The exogenous and endogenous ROS generation mediated by ApoE4 and Aβ can activate inflammatory signaling pathways, primarily through NF-κB [65, 88–90]. ApoE4 can also directly influence NF-κB transcription [57]. 2.Excessive ROS production and lysosomal damage (leakage of hydrolases) induced by ApoE4, Aβ, tau, and other factors can activate the NLRP3 inflammasome, promoting the cleavage of Pro-IL-1β and Pro-IL-18 [71, 90]. 3.TREM2 not only promotes phagocytosis, suppresses inflammation, and reduces lipid accumulation, but also supports aerobic glycolysis via the mTOR pathway to provide energy for the inflammatory response; however, its signaling may be negatively regulated by ApoE4 [39, 95, 98]. 4. ApoE4-mediated lipid metabolism dysregulation can also enhance MHC-II-dependent antigen presentation and T cell activation in microglia, while upregulating neuronal MHC-I expression, thereby amplifying immune signaling [129]. 5.Under inflammatory conditions, ApoE4 increases GLS activity, further inducing microglial activation and promoting glutamate production and release, exacerbating neurotoxicity [137]. ATP adenosine triphosphate; DAP12 DNAX-activating protein of 12 kDa; HMGB1 high mobility group box 1; Gln glutamine; GLS glutaminase; GLu glutamate; LilrB3 leukocyte immunoglobulin-like receptor B3; MHC major histocompatibility complex; MyD88 myeloid differentiation primary response gene 88; TLR4 toll-like receptor 4; TRAF2 nuclear factor receptor-associated factor 2.

Existing studies have shown that TREM2, one of the receptors for ApoE, plays a complex and dynamic role in the progression of AD. In the early stages, TREM2 activation may aid in clearing Aβ plaques and suppressing inflammation through the PI3K/NF-κB pathway, providing neuroprotective effects. However, as the disease advances, prolonged TREM2 activation fails to effectively clear Aβ and instead exacerbates inflammation [95, 96]. To explore whether ApoE4 affects TREM2-mediated phagocytosis of apoptotic cells, Kloske et al. activated phagocytic responses in mice by injecting phosphatidylserine (PS), a key marker on the membrane of apoptotic cells and a major activator of TREM2. The results showed that, compared to control mice, ApoE4 mice exhibited lower levels of microglial activation and weakened TREM2-mediated phagocytosis. However, these mice showed significantly upregulated pro-inflammatory gene expression, suggesting that ApoE4 may have a negative regulatory effect on TREM2 signaling [95]. In vitro experiments by Li et al. also supported this view [97]. TREM2 has also been confirmed to be a key transcriptional regulator of cholesterol transport and metabolism, and its functional deficiency leads to the accumulation of pathogenic lipids in microglia [98]. Conversely, ApoE4-mediated lipid metabolism dysregulation can inhibit TREM2 signaling, impairing the clearing function and anti-inflammatory capacity of microglia [99] (Fig. 3).

Astrocytes primarily function to provide metabolic support to neurons, but like microglia, they also play an important role in immune responses in the central nervous system and are considered key regulators of neuroinflammation in AD [100]. Studies show that when microglia are activated and produce IL-1β, C1q, and TNF-α, astrocytes respond and become activated, forming a positive feedback loop that releases inflammatory factors, thus creating a harmful environment for surrounding tissue [101]. Moreover, increasing evidence suggests that ApoE4-mediated lipid dysregulation also plays a significant role in the pro-inflammatory activation of astrocytes. Recent research by Sienski et al., using astrocytes derived from iPSCs of ApoE4 or ApoE3 carriers, indicated that compared to ApoE3-carrying astrocytes, ApoE4 promotes the accumulation of lipid droplets and the buildup of unsaturated fatty acids [102]. This ApoE4-dependent lipid droplet accumulation impairs the ability of astrocytes to support neuronal metabolism and synaptic function [103]. Furthermore, the accumulation of lipid droplets is sufficient to induce astrocyte reactivity, triggering the secretion of inflammatory chemokines and cytokines [99, 104]. These two types of glial cells cooperate to amplify neuroinflammatory signaling, leading to oxidative stress, iron overload, and neurotoxicity, which worsen neuronal damage [101, 104].

Based on the current understanding of the inflammatory mechanisms in AD, regulating the inflammatory response appears to be a promising therapeutic strategy for AD. Although many epidemiological studies support the idea that long-term use of nonsteroidal anti-inflammatory drugs (NSAIDs) can help prevent AD, these drugs do not slow the progression of the disease in diagnosed patients and may cause many adverse side effects during prolonged use [105]. One potential reason for their limited efficacy is the challenge of crossing the blood-brain barrier (BBB), a key factor influencing drug effectiveness. To address this, we have explored strategies to enhance drug delivery across the BBB. By synthesizing ApoE or ApoB amino acid fragments that bind to the low-density lipoprotein receptor (LRP) and conjugating them with our designed iron-chelating peptide J-bs5YP, we successfully transported the peptide into the brains of AD mice. This promoted Slc40a1 transcription and increased ferroportin levels, facilitating excess iron and free radical clearance, thereby reducing Aβ plaque formation, tau phosphorylation, and neuronal damage [106, 107]. Future advancements in optimizing BBB-targeting strategies and developing multifunctional drug designs could enhance therapeutic efficacy, offering new hope for AD treatment.

## The impact of ApoE4 on brain energy metabolism

The brain is a high-energy-consuming organ with extremely active metabolism. Under normal physiological conditions, the primary source of energy for the brain is glucose. Despite comprising only about 2% of body weight, the brain consumes approximately 20–25% of the body’s glucose, providing energy through glycolysis, the tricarboxylic acid cycle, and mitochondrial OXPHOS [108]. Impaired glucose metabolism in the brain is an early feature of AD. Before clinical symptoms appear in individuals with mild cognitive impairment (MCI) or AD, the brain already shows a decline in glucose metabolism, and there is evidence suggesting that ApoE4 may play a key role in this process [109, 110]. An 84-month longitudinal FDG-PET study showed that, in the context of MCI, the ApoE ε4 genotype is associated with a longitudinal decline in glucose uptake in multiple brain regions [109]. In a study involving 806 cognitively normal (CN) participants, 35 MCI patients, and 35 AD patients, ApoE ε4 carriers exhibited significantly lower glucose metabolism in multiple brain regions, including those characteristic of AD, compared to non-carriers. In particular, the decline in glucose metabolism was especially pronounced in AD patients carrying ApoE ε4 [110]. These findings underscore the potential importance of the ApoE ε4 gene’s influence on glucose metabolism in the pathophysiology of AD. Although the exact mechanisms remain unclear, existing studies suggest that the effects may be linked to impaired insulin receptor signaling [28, 111]. Decreased expression of key glycolytic enzymes such as hexokinase and glucose transporters [28, 112, 113]. PPAR-γ and PGC-1α are two crucial regulators of energy metabolism, and they work synergistically to play a critical role in maintaining the bioenergetic homeostasis of the brain [112]. The PPAR-γ/PGC-1α signaling pathway has been shown to be inhibited in the brains of ApoE4 carriers, and overexpression of PGC-1α improves the defects in glycolysis and mitochondrial respiration induced by ApoE4 [112]. PPAR-γ agonists, such as Thiazolidinediones (TZDs), can improve insulin sensitivity and glucose metabolism, potentially offering benefits for AD patients [114]. As the most abundant glial cells in the brain, astrocytes play a key role in providing metabolic substrates to neurons, including lactate, lipids, amino acids, and ketone bodies [115]. Astrocytes are also the only cell type in the brain that stores glycogen, and they can sense synaptic activity and energy demands. During energy crises, they convert glycogen into lactate and shuttle it to neurons as a metabolic fuel, a process known as the astrocyte-neuron lactate shuttle (ANLS) [116]. By relying on lactate produced by astrocytes, neurons are able to maintain a high rate of OXPHOS while ensuring sufficient glucose enters the pentose phosphate pathway to generate NADPH, which is crucial for maintaining their antioxidant status and normal function [117]. It has been reported that in young adult ApoE4 carriers, the expression of MCT4, a key transporter involved in astrocytic lactate secretion, is reduced, while the expression of MCT2, a lactate transporter in neurons, is upregulated. The abnormal expression of these two key ANLS components suggests that the ANLS may be disrupted by ApoE4 [113]. A study by Lee et al. found that astrocytes from ApoE4 carriers exhibit significant mitochondrial respiratory dysfunction, while their glycolytic activity is markedly increased [118]. Excessive glycolysis in astrocytes could lead to the production of more lactate, which may acidify the extracellular environment and impact the function of other cells, including promoting Aβ aggregation [118, 119]. Additionally, mitochondrial dysfunction in ApoE4 astrocytes may limit metabolite supply and accumulate oxidative stress, triggering AD-related pathological changes [118]. Although fatty acids are not the primary energy source for the brain, in situations of glucose and glycogen depletion, fatty acids and ketone bodies provided by astrocytes become important alternative energy sources. In addition, fatty acids provide essential structural support for neurons and play a significant role in antioxidant protection, injury repair, and signal transduction regulation [120]. However, due to the limited ability of neurons to oxidize fatty acids, this can lead to elevated levels of free fatty acids (FFAs) and lipid peroxidation [121]. As a result, neurons need to transfer oxidized fatty acids to nearby glial cells to avoid the toxic effects of lipid peroxides [102, 122]. In the lipid transport process between neurons and glial cells, fatty acid transport proteins (FATPs) and ApoE play key roles. The absence of FATPs and the presence of ApoE4 both impair lipid transport [103, 122, 123]. A study by Qi et al. on primary embryonic neurons from the hippocampus of humanized ApoE3 and ApoE4 knockin mice revealed that damage to lipid turnover caused by ApoE4 leads to elevated FA levels in neurons, which subsequently suppresses neuronal glucose metabolism and mitochondrial OXPHOS. At the same time, FA output, as well as subsequent uptake and degradation by astrocytes, is impaired, leading to the accumulation of lipid droplets (LDs) in astrocytes. The mitochondrial function and aerobic glycolysis in astrocytes are suppressed, reducing their metabolic and structural support for neurons, which in turn affects neuronal axonal growth and synaptic density [103]. Based on the tight coupling of lipid metabolism between glial cells and neurons, abnormal lipid metabolism in glial cells inevitably leads to changes in the composition and proportion of neuronal membrane lipids, thereby affecting the efficiency of synaptic signaling [124]. A study by Miranda et al. demonstrated that in neurons treated with conditioned medium from ApoE4 astrocytes, several lipids associated with endosomal-lysosomal trafficking accumulated in a dose-dependent manner, leading to significant alterations in neuronal membrane lipid composition. Levels of the primary membrane phospholipids, phosphatidylcholines (PC) and ether phosphatidylcholines (PCe), decreased, while levels of phosphatidic acid (PA), which is associated with neurotransmitter vesicle cycling, increased. This has been suggested as a potential mechanism underlying the hyperactivity of ApoE4 neurons [125]. Neuronal hyperactivity is an early phenotype of human AD and may play a direct role in disease pathogenesis [126]. Additionally, since PCe species are natural scavengers of reactive oxygen species, their reduction may weaken the antioxidant protection of neurons [125].

**Fig. 4:**
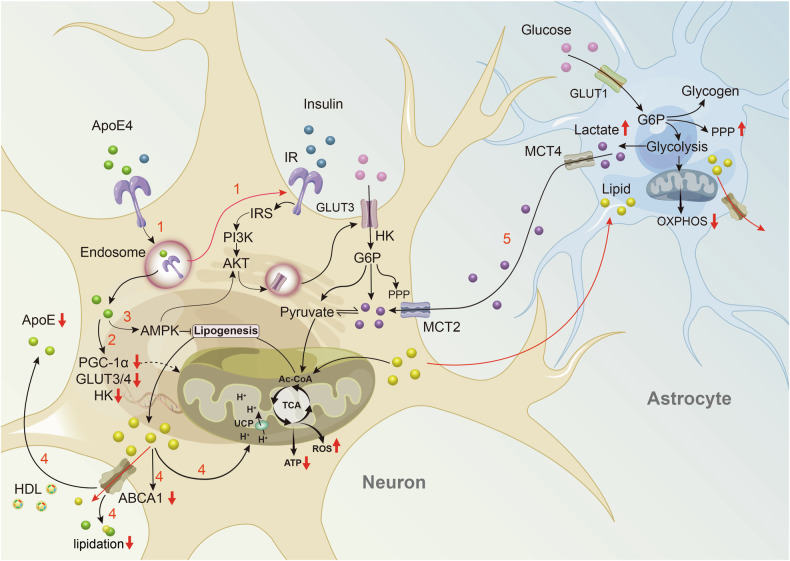
Possible mechanisms of ApoE4 in neuronal energy metabolism dysregulation. 1. ApoE4 competes with insulin for the IR and traps the IR in the endosome, thereby hindering the endocytic recycling of IR [111]. 2. ApoE4 inhibits key molecules in the insulin signaling pathway, including the HK, GLUT, and PGC-1α, the master regulator of mitochondrial biogenesis, leading to impaired insulin signaling, glycolytic function, and mitochondrial respiration [28, 112, 138]. 3. Under specific conditions (such as low ATP and high FAs levels), AMPK may be activated in ApoE4-expressing neurons to adapt to and mitigate fatty acid toxicity [103]. 4. ApoE4-mediated cholesterol accumulation may interfere with ABCA1 localization or recycling at the plasma membrane, impairing its lipid efflux function and disrupting ApoE lipidation. ApoE levels may also be affected, and inadequate cholesterol transport leads to further intracellular lipid accumulation [139–141]. Excess fatty acids (FAs) can cause mitochondrial uncoupling and dysfunction, further inhibiting glucose metabolism and mitochondrial OXPHOS in neurons [103]. 5. Lipid degradation in astrocytes is also impaired due to mitochondrial dysfunction, leading to lipid accumulation and reduced ability to receive additional lipid transfer from neurons. Aerobic glycolysis is suppressed, resulting in excessive lactate production [103, 118, 119]. Although lactate shuttling can provide metabolic fuel to neurons, this compromised metabolic state cannot effectively support sustained and stable neuronal metabolism, and excessive lactate accumulation may further damage the surrounding environment [118, 119]. Red lines indicate blocked pathways. Ac-CoA acetyl-CoA, ABCA1 ATP-binding cassette transporter A1, FAs fatty acids, G6P glucose-6-Phosphate, GLUT glucose transporter, HDL high density lipoprotein, HK hexokinase, IR insulin receptor, IRS insulin receptor substrate, MCT monocarboxylate Transporter, PGC-1α peroxisome proliferator-activated receptor gamma coactivator 1 alpha, PPP Pentose Phosphate Pathway, TCA tricarboxylic Acid Cycle, UCP uncoupling Protein.

A study by Victor et al. showed that ApoE4 impairs the lipid uptake ability of microglia, leading to cholesterol accumulation in specific regions of the neuronal cell membrane. This enhances the activity of lipid-gated G-protein-gated inwardly rectifying K+ channels, resulting in hyperpolarization of the neuronal resting membrane potential and reduced neuronal excitability—a phenomenon commonly observed in later stages of AD [127]. Oligodendrocytes, the myelinating cells of the central nervous system, are also affected by ApoE4. Studies have shown that in both human and mouse oligodendrocytes, ApoE4 disrupts cholesterol synthesis and its transport to the plasma membrane, leading to increased intracellular cholesterol accumulation and endoplasmic reticulum stress, which impacts myelination and neuronal synaptic signaling. Treatment with cyclodextrin to promote cholesterol transport has been shown to enhance axonal myelination in ApoE4 mice, improving learning and memory [128]. These findings support the role of ApoE4-mediated lipid metabolic dysregulation in AD pathology and highlight the therapeutic potential of lipid regulation in AD treatment (Fig. 4).

## Summary and perspectives

Despite over a century of research, the etiology of late-onset Alzheimer’s disease (LOAD) remains elusive, and effective treatments continue to be a major medical challenge. ApoE4, the strongest genetic risk factor for AD, plays a central role in Aβ deposition, tau tangles, neuroinflammation, and various downstream pathways. Thus, targeting ApoE4 offers a promising therapeutic strategy for AD. Current approaches focus on: (1) blocking ApoE4-Aβ interactions, (2) targeting ApoE4 receptors, (3) enhancing ApoE4 lipidation, (4) developing ApoE4 antibodies, and (5) gene therapy to correct the ApoE4 genotype or function. While most research remains at the preclinical stage, significant progress has been made, particularly in gene therapy. Several teams are currently conducting clinical trials using ApoE2-expressing constructs (e.g., LX1001) to treat AD patients carrying the ApoE4 allele, with the goal of converting the ApoE genotype from ApoE4 to ApoE2. The Phase I safety study (NCT03634007) has been completed, and the trial is now progressing into a longer-term follow-up study (NCT05400330, for further details see: clinicaltrials.gov).

While substantial progress has been made, several challenges remain that hinder further advancement. First, AD’s complex, heterogeneous etiology makes targeting a single pathway unlikely to yield significant results. Second, the exact mechanisms by which ApoE4 contributes to AD, whether through a toxic gain-of-function or loss of protective function, are not yet fully understood. Nonetheless, ApoE4 remains a promising target for therapy.

Future research should utilize large-scale genomic technologies to deepen our understanding of ApoE4’s role in AD pathology and to identify new therapeutic targets. Enhanced interdisciplinary collaboration, integrating basic research, clinical trials, and translational medicine, will accelerate the development of ApoE4-related therapies. Emerging technologies like artificial intelligence and big data analysis will help clarify the complex relationship between ApoE4 and AD, paving the way for personalized treatments tailored to specific genotypes. Long-term studies are essential to evaluate the efficacy and safety of new therapies for ApoE4 carriers.

## Data Availability

Data sharing not applicable to this article as no datasets were generated or analyzed during this study.
